# Supplementary material for: Fusarium sacchari hypovirus 1, a Member of Hypoviridae with Virulence Attenuation Capacity in Phytopathogenic Fusarium Species
Source: Viruses. 2024 Apr 15;16(4):608. doi: 10.3390/v16040608 (PMC11054305; doi:10.3390/v16040608)
Supplement: Supplementary file 1 [file viruses-16-00608-s001.zip › Supplementary figure-FsHV1.pdf]

Supplementary Figure S1.

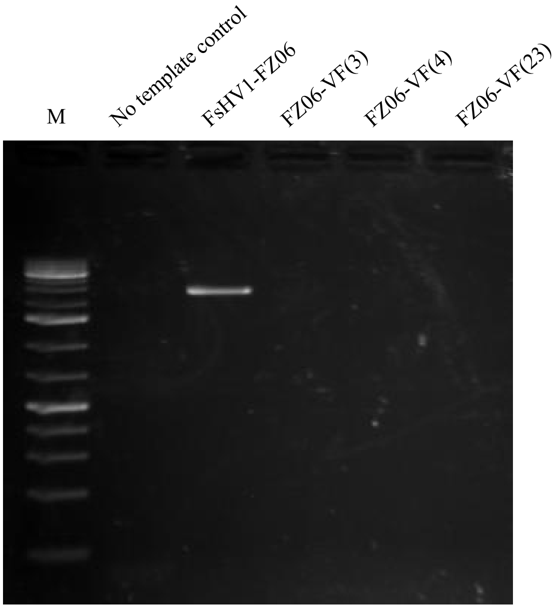

Supplementary Figure S1. Detection of detoxified strain FZ06-VF. RT-PCR detection of virus-free strains FZ06-VF by specific primer pairs FsHV1-DefectF/R. Lane M，GeneRuler 1 kb Plus DNA Ladder; FsHV1-FZ06 as a positive control.

Supplementary Figure S2.

Fusarium sacchari

Fusarium andiyazi

Fusarium solani

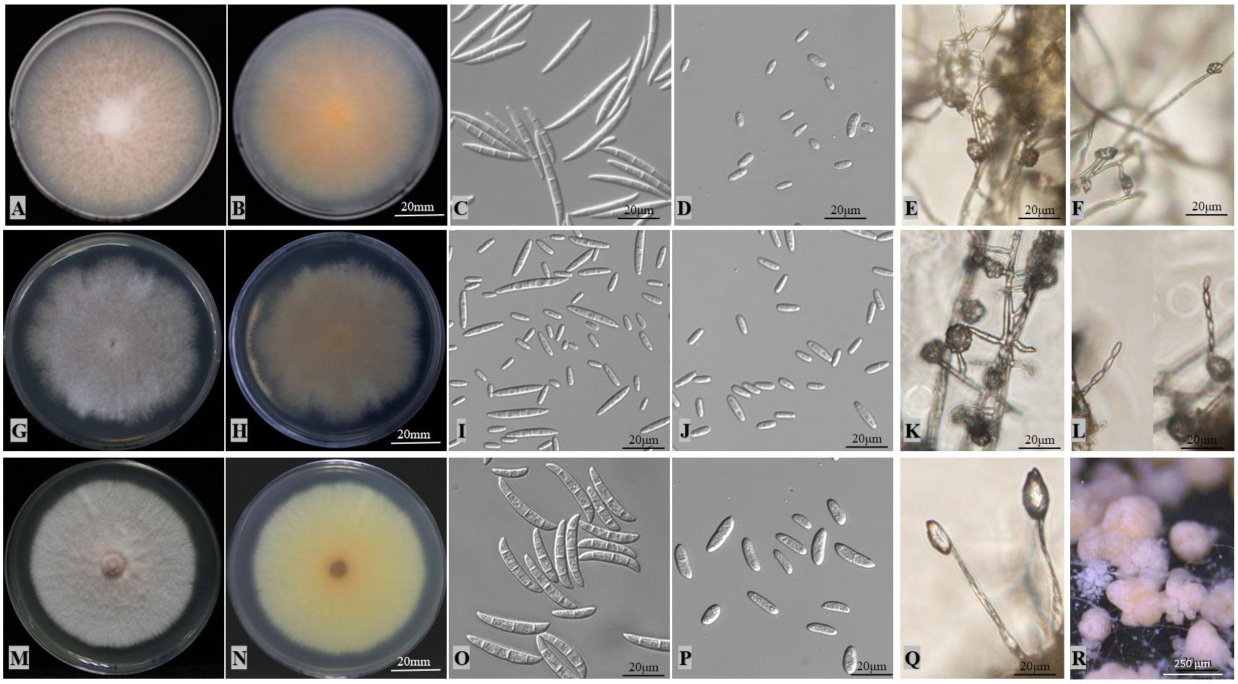

Supplementary Figure S2.Morphological characterization of *Fusarium sacchari*, *Fusarium andiyazi* and *Fusarium solani*.(A-F):Morphological characterization of Fusarium sacchari.(A and B)Fungal colony growing on the PDA.(C)Macroconidia.(D)Microconidia.(E-F)Microconidia formed on short aerial conidiophores arising from hyphae on CLA.(G-L)Morphological characterization of Fusarium andiyazi.(G and H)Fungal colony growing on the PDA.(I)Macroconidia.(J)Microconidia.(K)Microconidia in situ on CLA.(L)Microconidial chains.(M-R)Morphological characterization of Fusarium andiyazi.(M and N)Fungal colony growing on the PDA.(O)Macroconidia.(P)Microconidia.(Q and R)Microconidia in situ on CLA.A,B,G,H,M,N,scale bar = 25mm.C-F,I-L,O-R,scale bar = 25μm.

Supplementary Figure S3.

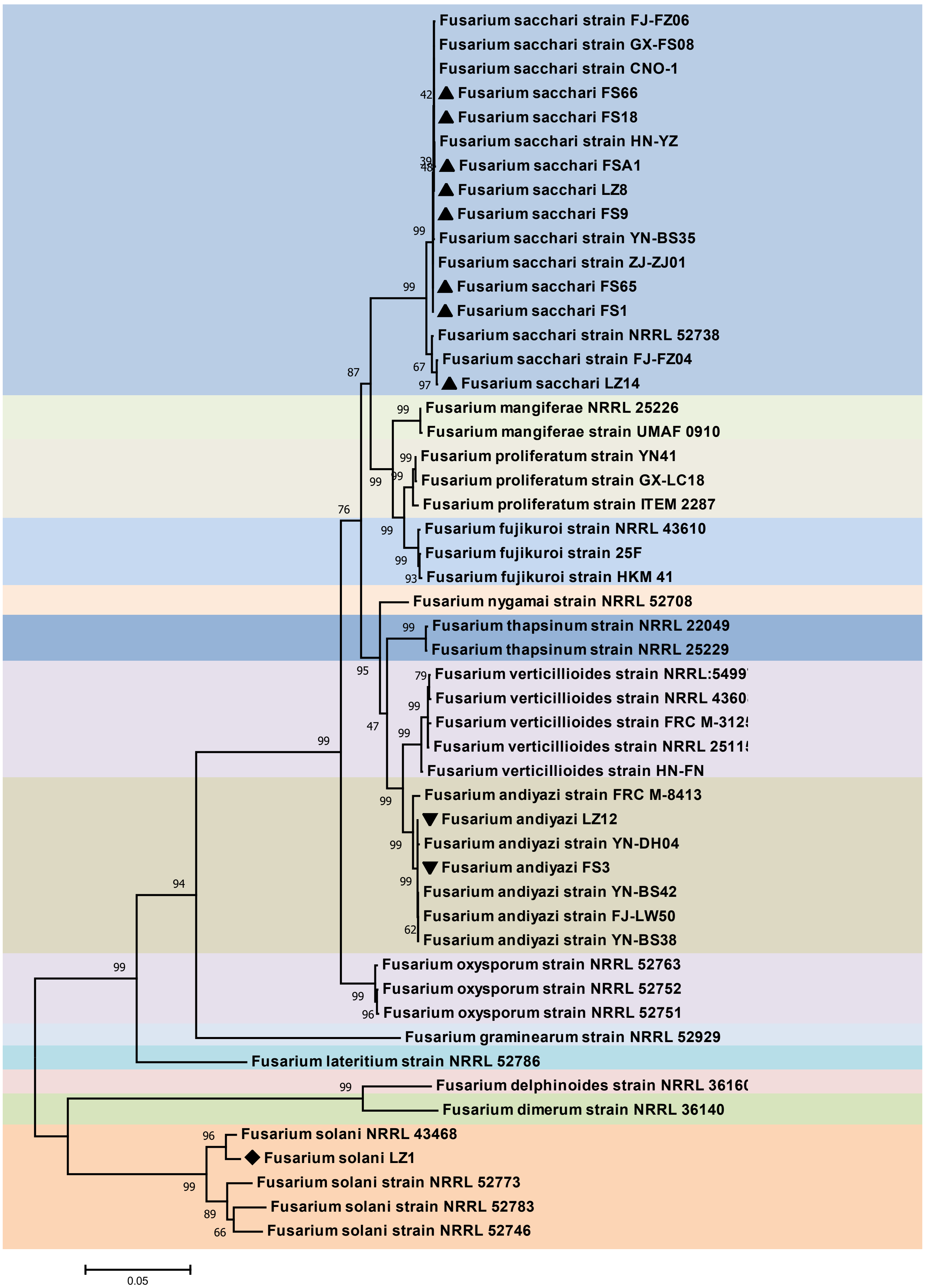

Supplementary Figure S3. Phylogenetic analysis of Fusarium. Phylogenetic tree inferred by Maximum Likelihood using TEF-1α+RPB1+RPB2 gene sequence with the reference sequences from other Fusarium species,including *F.sacchari*、*F.mangiferae*、*F.proliferatum*、*F.fujikuroi*、*F.nygamai*、*F.thapsinum*、*F.verticillioides*、*F.andiyazi*、*F.oxysporum*、*F.graminearum*、*F.lateritium*、*F.delphinoides*、*F.dimerum*、*F.solani.* Phylogenetic reconstruction was performed by bootstrap test (1000 replicates) showing the relationship between the 12 Fusarium spp. isolates identified in this study.

Supplementary Figure S4.

A

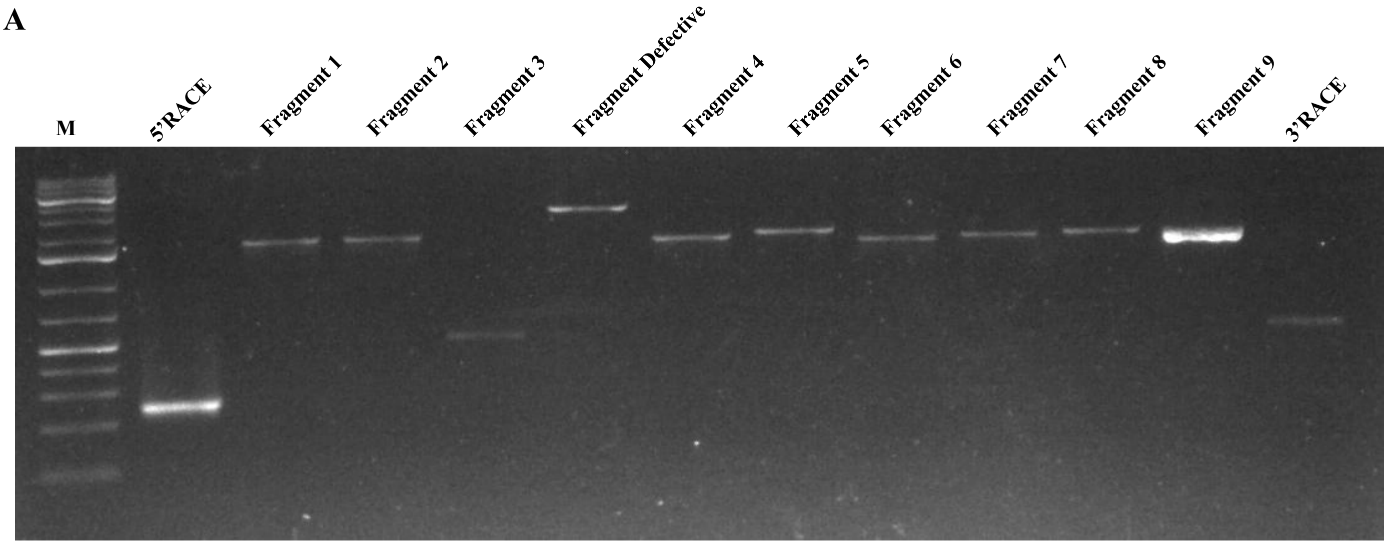

B

Full-length nucleotide sequence similarity

	FsHV1-FZ06	FsHV1-FS1	FsHV1-FS9	FsHV1-FS65	FsHV1-FS66	FsHV1-LZ8	FsHV1-FSA1	FsHV1-FS3	FsHV1-FS18	FsHV1-LZ1	FsHV1-LZ12	FsHV1-LZ14
FsHV1-FZ06	-	99.9%	99.4%	99.5%	99.4%	99.0%	99.0%%	99.7%	99.2%	99.2%	98.9%	99.2%
FsHV1-FS1		-	99.3%	99.5%	99.2%	99.0%	98.9%	99.6%	99.1%	99.1%	98.8%	99.1%
FsHV1-FS9			-	99.6%	99.2%	99.1%	99.0%	99.2%	99.1%	99.1%	98.8%	99.2%
FsHV1-FS65				-	99.4%	99.1%	99.2%	99.4%	99.2%	99.1%	98.9%	99.3%
FsHV1-FS66					-	99.0%	98.9%	99.3%	99.0%	99.1%	98.6%	99.0%
FsHV1-LZ8						-	98.8%	99.2%	99.2%	99.7%	98.9%	99.6%
FsHV1-FSA1							-	98.9%	98.9%	99.0%	99.1%	98.9%
FsHV1-FS3								-	99.2%	99.1%	98.8%	99.1%
FsHV1-FS18									-	99.3%	99.0%	99.7%
FsHV1-LZ1										-	99.1%	99.0%
FsHV1-LZ12											-	99.0%
FsHV1-LZ14												-

C

Amino acid sequence similarity

	FsHV1-FZ06	FsHV1-FS1	FsHV1-FS9	FsHV1-FS65	FsHV1-FS66	FsHV1-LZ8	FsHV1-FSA1	FsHV1-FS3	FsHV1-FS18	FsHV1-LZ1	FsHV1-LZ12	FsHV1-LZ14
FsHV1-FZ06	-	99.90%	99.30%	99.40%	99.30%	99.30%	99.10%	99.80%	99.50%	99.40%	99.00%	99.30%
FsHV1-FS1			99.20%	99.30%	99.20%	99.20%	99.0%	99.70%	99.40%	99.30%	98.90%	99.20%
FsHV1-FS9				99.60%	99.20%	99.10%	99.20%	99.40%	99.40%	99.30%	98.90%	99.30%
FsHV1-FS65					99.30%	99.30%	99.40%	99.30%	99.60%	99.50%	99.10%	99.50%
FsHV1-FS66						99.00%	99.10%	99.20%	99.20%	99.10%	98.70%	99.10%
FsHV1-LZ8							99.0%	99.20%	99.40%	99.80%	99.00%	99.70%
FsHV1-FSA1								99.0%	99.20%	99.10%	98.9%	99.10%
FsHV1-FS3									99.30%	99.30%	98.90%	99.30%
FsHV1-FS18										99.60%	99.10%	99.60%
FsHV1-LZ1											99.10%	99.80%
FsHV1-LZ12												99.10%
FsHV1-LZ14												-

Supplementary Figure S4. Full-length amplification and sequence homology of 12 FsHV1 isolates. (**A**) Full-length amplification of FsHV1, including 12 fragments. Lane M， GeneRuler 1 kb Plus DNA Ladder. (**B**)The percent identity analysis between 12 FsHV1 full-length nucleotide sequence.(**C**)The percent identity analysis between 12 FsHV1 full-length nucleotide sequence.

Supplementary Figure S5.

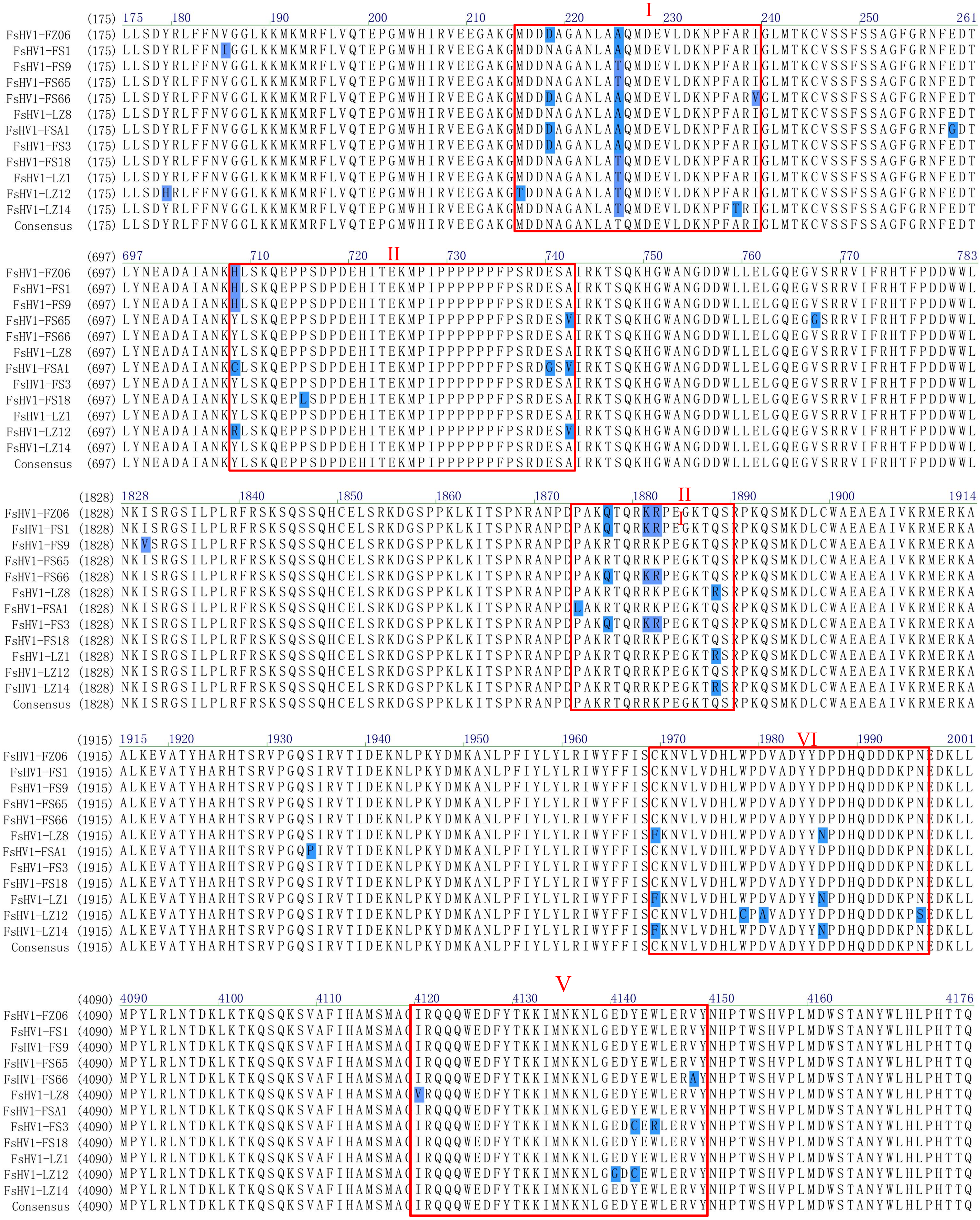

Supplementary Figure S5. Amino acid sequence alignment of 12 FsHV1 isolates. The red boxed regions I~V with more differences in amino acids.

Supplementary Figure S6.

A

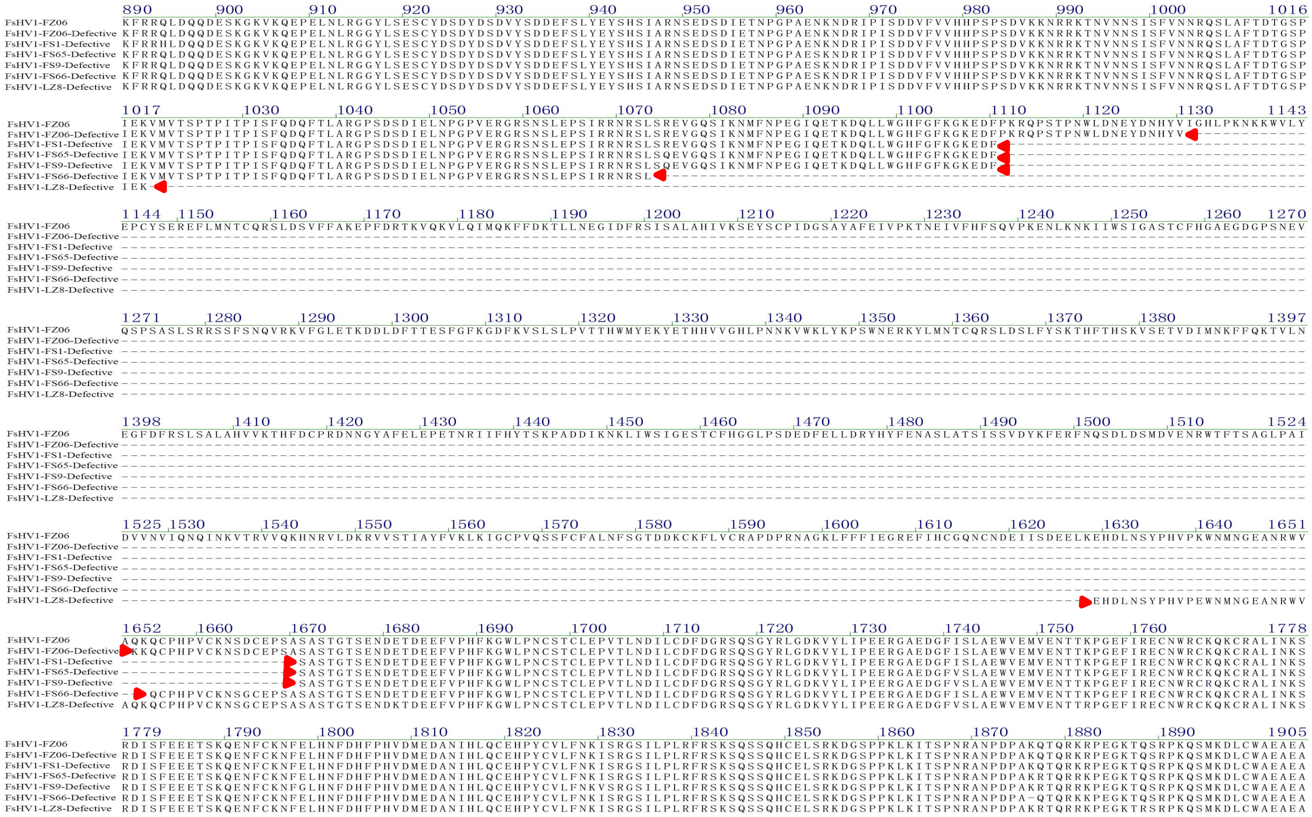

B

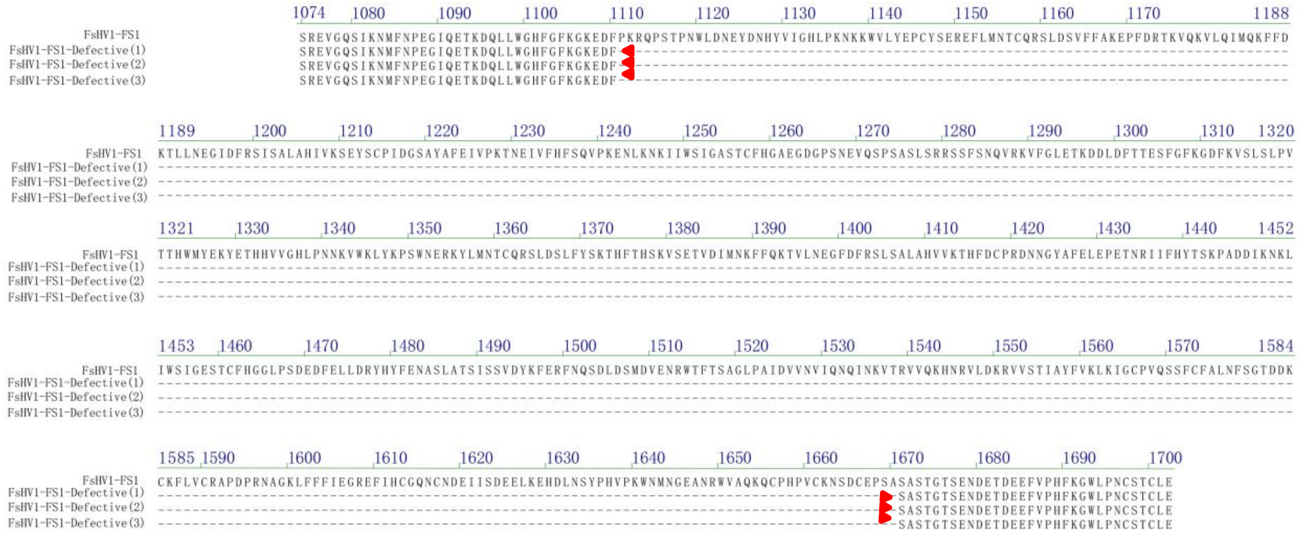

Supplementary Figure S6. The positional of amino acid sequences of FsHV1-Defective RNA. (**A**) Amino acid sequence alignment of the region corresponding between FsHV1-FZ06 and 6 FsHV1-Defective RNA. **------**， regions that are absent in 6 FsHV1 RNA. (**B**) Amino acid sequence alignment of the region corresponding between the same strain.

Supplementary Figure S7.

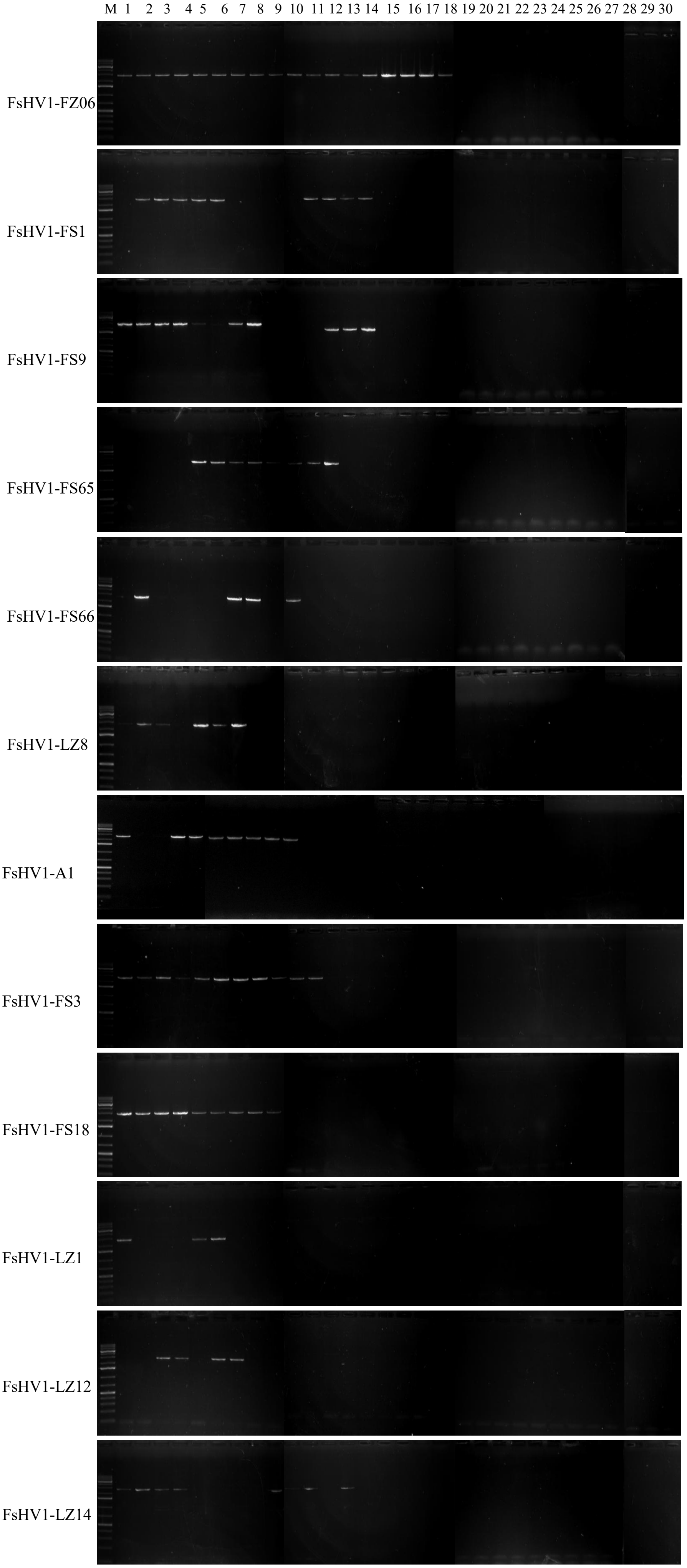

Supplementary Figure S7. Detection of FsHV1 after mycelial fusion. The target size of intact virus are 2500bp or the target size of defective virus about 800bp. Lane M， GeneRuler 1 kb Plus DNA Ladder .1~30, each of the 12 FsHV1 isolates horizontal transmission to the FZ06-VF strain.
