# Supplementary material for: Fusarium sacchari hypovirus 1, a Member of Hypoviridae with Virulence Attenuation Capacity in Phytopathogenic Fusarium Species
Source: Viruses. 2024 Apr 15;16(4):608. doi: 10.3390/v16040608 (PMC11054305; doi:10.3390/v16040608)
Supplement: Supplementary file 1 [file viruses-16-00608-s001.zip › Supplementary table.pdf]

Supplementary Table S1. The *Fusarium*-infecting mycoviruses.

No.	Genus	Genome type	Mycovirus	Accession no.
**1**	*Chrysoviridae*	dsRNA	Fusarium graminearum mycovirus China 9(FgV-China 9)	dsRNA1:HQ282213；dsRNA2:HQ2228214；dsRNA3:HQ282215；dsRNA4:HQ2228216；dsRNA5:HQ282217
**2**	*Chrysoviridae*	dsRNA	Fusarium graminearum dsRNA mycovirus 2(FgV2)	dsRNA1:HQ343295；dsRNA2:HQ343296；dsRNA3:HQ3343297；dsRNA4:HQ343298；
**3**	*Chrysoviridae*	dsRNA	Fusarium oxysporum f. sp. dianthi mycovirus 1(FodV)	dsRNA1:KP876629；dsRNA2:KP876630；dsRNA3:KP876631；dsRNA4:KP876632
**4**	*Chrysoviridae*	dsRNA	Fusarium sacchari chrysovirus 1(FsCV1)	dsRNA1: MN295964; dsRNA2: MN295965; dsRNA3: MN295966; dsRNA4: MN295967
**5**	*Chrysoviridae*	dsRNA	Fusarium oxysporum chrysovirus 1(FoCV1)	dsRNA1:EF152346；dsRNA2:EF152347；dsRNA3:EF152348
**6**	*Megabirnaviridae*	dsRNA	Fusarium pseudograminearum megabirnavirus 1(FpgMBV1)	RNA1: MH057692 RNA2: MH057693
**7**	*Partitiviridae*	dsRNA	Fusarium solani virus 1(FsV1)	RNA1: NC_003885

				RNA2: NC_003886
**8**	*Partitiviridae*	dsRNA	Fusarium poae virus 1(FpV1)	RNA1: NC_003883<br>RNA2: NC_003884
**9**	*Partitiviridae*	dsRNA	Fusarium solani partitivirus 2(FsPV2)	LC006130
**10**	*Partitiviridae*	dsRNA	Fusarium oxysporum f. sp. Cubense partitivirus 1 (FocPV1)	OQ685981
**11**	*Partitiviridae*	dsRNA	Fusarium oxysporum f. sp. cubense partitivirus 2 (FocPV2)	OQ685986
**12**	*Partitiviridae*	dsRNA	Fusarium oxysporum f. sp. cubense partitivirus 3 (FocPV3)	OQ685982
**13**	*Partitiviridae*	dsRNA	Fusarium oxysporum f. sp. cubense partitivirus 4 (FocPV4)	OQ685980
**14**	*Partitiviridae*	dsRNA	Fusarium oxysporum f. sp. cubense partitivirus 5 (FocPV5)	OQ685983
**15**	*Partitiviridae*	dsRNA	Fusarium oxysporum f. sp. cubense partitivirus 6 (FocPV6)	OQ685984
**16**	*Partitiviridae*	dsRNA	Fusarium oxysporum f. sp. cubense partitivirus 7 (FocPV7)	OQ685985
**17**	*Partitiviridae*	dsRNA	Fusarium mangiferae partitivirus 1 (FmPV1)	segment 1:MZ493897<br>segment 2:MZ493898
**18**	*Partitiviridae*	dsRNA	Fusarium mangiferae partitivirus 2 (FmPV2)	segment 1:MZ493899<br>segment 2:MZ493800
**19**	*Polymycoviridae*	dsRNA and ssRNA	Fusarium redolens polymycovirus 1(FrPmV1)	RNA1:MK609920<br>RNA2:MK609921<br>RNA3:MK609922<br>RNA4:MK609923<br>RNA5:MK609924<br>RNA6:MK609925<br>RNA7:MK609926<br>RNA8:MK609927
**20**	*Totiviridae*	dsRNA	Fusarium asiaticum victorivirus 1(FaVV1)	MH615042
**21**	*Alternaviridae*	dsRNA	Fusarium graminearum alternavirus 1(FgAV1)	MG254901
**22**	*Alternaviridae*	dsRNA	Fusarium incarnatum alternavirus 1(FiAV1)	MH899114

**23**	*Fusagraviridae*	dsRNA	Fusarium poae dsRNA virus 2(FpV2)	KU728180
**24**	*Fusagraviridae*	dsRNA	Fusarium poae dsRNA virus 3(FpV3)	KU728181
**25**	*Fusagraviridae*	dsRNA	Fusarium virguliforme dsRNA mycovirus 1(FvV1)	JN671444
**26**	*Fusagraviridae*	dsRNA	Fusarium virguliforme dsRNA mycovirus 2(FvV2)	JN671443
**27**	*Fusagraviridae*	dsRNA	Fusarium graminearum dsRNA mycovirus-3(FgV3)	NC_013469
**28**	*Curvulaviridae*	dsRNA	Fusarium graminearum dsRNA mycovirus-4(FgV4)	RNA1: NC_013470<br>RNA2: NC_013471
**29**	*Curvulaviridae*	dsRNA	Fusarium graminearum dsRNA mycovirus-5(FgV5)	RNA1: KX380787<br>RNA2: KX380788
**30**	*Deltaflexiviridae*	+ ssRNA	Fusarium graminearum deltaflexivirus 1(FgDFV1)	KX015962
**31**	*Endornaviridae*	+ ssRNA	Fusarium oxysporum f. sp. cubense endornavirus 1 (FocEV1)	OQ685975
**32**	*Botourmiaviridae*	+ssRNA	Fusarium oxysporum f. sp. cubense ourmia-like virus 1 (FocOLV1)	OQ685967
**33**	*Botourmiaviridae*	+ssRNA	Fusarium oxysporum f. sp. cubense ourmia-like virus 2 (FocOLV2)	OQ685968
**34**	*Botourmiaviridae*	+ssRNA	Fusarium oxysporum f. sp. cubense ourmia-like virus 3 (FocOLV3)	OQ685970
**35**	*Botourmiaviridae*	+ssRNA	Fusarium oxysporum f. sp. cubense ourmia-like virus 4 (FocOLV4)	OQ685969
**36**	*Botourmiaviridae*	+ssRNA	Fusarium asiaticum ourmiavirus 2(FaOV2)	RNA1:MZ969060<br>RNA1:MZ969061
**37**	*Botourmiaviridae*	+ssRNA	Fusarium asiaticum ourmiavirus 1(FaOV1)	MZ969063
**38**	*Botourmiaviridae*	+ssRNA	Fusarium mangiferae botourmiavirus 1(FmBOV1)	MZ493907
**39**	*Botourmiaviridae*	+ssRNA	Fusarium mangiferae botourmiavirus 2(FmBOV2)	MZ493908
**40**	*Botourmiaviridae*	+ssRNA	Fusarium mangiferae botourmiavirus 3(FmBOV3)	MZ493909

**41**	*Hypoviridae*	+ ssRNA	Fusarium graminearum hypovirus 1(FgHV1)	KC330231
**42**	*Hypoviridae*	+ ssRNA	Fusarium graminearum hypovirus 2(FgHV2)	KP208178
**43**	*Hypoviridae*	+ ssRNA	Fusarium langsethiae hypovirus 1(FlHV1)	KY120321
**44**	*Hypoviridae*	+ ssRNA	Fusarium sacchari hypovirus 1(FsHV1)	MN295969
**45**	*Hypoviridae*	+ ssRNA	Fusarium sambucinum hypovirus 1 (FsamHV1)	NC_076613
**46**	*Narnaviridae*	+ ssRNA	Fusarium circinatum mitovirus 1(FcMV1)	KF803546
**47**	*Narnaviridae*	+ ssRNA	Fusarium globosum mitovirus 1(FgMV1)	LC006128
**48**	*Narnaviridae*	+ ssRNA	Fusarium boothii mitovirus 1(FbMV1)	LC425112
**49**	*Narnaviridae*	+ ssRNA	Fusarium andiyazi mitovirus 1(FaMV1)	MN295972
**50**	*Narnaviridae*	+ ssRNA	Fusarium andiyazi mitovirus 2(FaMV2)	MN295975
**51**	*Narnaviridae*	+ ssRNA	Fusarium sacchari mitovirus 1(FsMV1)	MN295976
**52**	*Narnaviridae*	+ ssRNA	Fusarium poae narnavirus 1(FpNV1)	NC_030865
**53**	*Narnaviridae*	+ ssRNA	Fusarium asiaticum narnavirus 1	MZ969062
**54**	*Mitoviridae*	+ ssRNA	Fusarium coeruleum mitovirus 1(FocMV1)	LC006129
**55**	*Mitoviridae*	+ ssRNA	Fusarium oxysporum f. sp. cubense mitovirus 1 (FocMV1)	OQ685973
**56**	*Mitoviridae*	+ ssRNA	Fusarium oxysporum f. sp. cubense mitovirus 2 (FocMV2)	OQ685971
**57**	*Mitoviridae*	+ ssRNA	Fusarium oxysporum f. sp. cubense mitovirus 3 (FocMV3)	OQ685974
**58**	*Mitoviridae*	+ ssRNA	Fusarium oxysporum f. sp. cubense mitovirus 4 (FocMV4)	OQ685972
**59**	*Mitoviridae*	+ ssRNA	Fusarium asiaticum mitovirus 1	MZ969051
**60**	*Mitoviridae*	+ ssRNA	Fusarium asiaticum mitovirus 2	MZ969052
**61**	*Mitoviridae*	+ ssRNA	Fusarium asiaticum mitovirus 3	MZ969053
**62**	*Mitoviridae*	+ ssRNA	Fusarium asiaticum mitovirus 4	MZ969054
**63**	*Mitoviridae*	+ ssRNA	Fusarium asiaticum mitovirus 5	MZ969055
**64**	*Mitoviridae*	+ ssRNA	Fusarium asiaticum mitovirus 6	MZ969056
**65**	*Mitoviridae*	+ ssRNA	Fusarium asiaticum mitovirus 7	MZ969057

**66**	*Mitoviridae*	+ ssRNA	Fusarium asiaticum mitovirus 8	MZ969058
**67**	*Mitoviridae*	+ ssRNA	Fusarium poae mitovirus 1	MZ969059
**68**	*Mitoviridae*	+ ssRNA	Fusarium mangiferae mitovirus 1 (FmMV1)	MZ493901
**69**	*Mitoviridae*	+ ssRNA	Fusarium mangiferae mitovirus 2 (FmMV2)	MZ493902
**70**	*Mitoviridae*	+ ssRNA	Fusarium mangiferae mitovirus 3 (FmMV3)	MZ493903
**71**	*Mitoviridae*	+ ssRNA	Fusarium mangiferae mitovirus 4 (FmMV4)	MZ493904
**72**	*Mitoviridae*	+ ssRNA	Fusarium mangiferae mitovirus 5 (FmMV5)	MZ493905
**73**	*Mitoviridae*	+ ssRNA	Fusarium mangiferae mitovirus 6 (FmMV6)	MZ493906
**74**	*Mitoviridae*	+ ssRNA	Fusarium verticillioides mitovirus 1 (FvMV1)	NC_076900
**75**	*Mitoviridae*	+ ssRNA	Fusarium andiyazi mitovirus 1 (FaMV1-162)	NC_076901
**76**	*Fusariviridae*	+ ssRNA	Fusarium asiaticum fusarivirus 1	MZ969065
**77**	*Fusariviridae*	+ ssRNA	Fusarium graminearum dsRNA mycovirus-1(FgV1)	NC_006937
**78**	*Fusariviridae*	+ ssRNA	Fusarium poae fusarivirus 2	OK524195
**79**	*Fusariviridae*	+ ssRNA	Fusarium pseudograminearum fusarivirus 1	OP100308
**80**	*Tymoviridae*	+ ssRNA	Fusarium graminearum mycotymovirus 1(FgMTV1)	KT360947
**81**	*Hadakaviridae*	+ ssRNA	Hadaka virus 1 (HadV1)	segment 1：LC519840<br>segment 2：LC519841<br>segment 3：LC519842<br>segment 4：LC519843<br>segment 5：LC519844<br>segment 6：LC519845<br>segment 7：LC519846<br>Segmen t8：LC519847<br>segment 9：LC519848<br>segment 10：LC519849<br>segment 11：LC519850

**82**	*Mymonaviridae*	- ssRNA	Fusarium graminearum negative-stranded RNA virus 1(FgNSRV-1)	MF276904
**83**	*Mymonaviridae*	-ssRNA	Fusarium oxysporum f. sp. cubence mymonavirus 1 (FocMyV1)	OQ685977
**84**	*Mymonaviridae*	-ssRNA	Fusarium oxysporum f. sp. cubense negative-stranded RNA virus 1 (FocNSRV1)	OQ685978
**85**	*Phenuiviridae*	-ssRNA	Fusarium sibiricum coguvirus 1(FsCV1)	OQ295988
**86**	*Phenuiviridae*	-ssRNA	Fusarium asiaticum mycobunyavirus 1 (FaMBV1)	segment L:MZ969068<br>segment M:MZ969069<br>segment S:MZ969070
**87**	unassigned	+ ssRNA	Fusarium oxysporum f. sp. cubense alphavirus-like virus (FocALV)	OQ685976
**88**	unassigned	-ssRNA	Fusarium oxysporum f. sp. cubense negative-stranded RNA virus 2 (FocNSRV2)	OQ685979
**89**	unassigned	+ ssRNA	Fusarium boothii large flexivirus 1(FbLFV1)	LC425115
**90**	unassigned	+ ssRNA	Fusarium sacchari alphavirus-like virus 1(FsALV1)	MN295968
**91**	unassigned	-ssRNA	Fusarium asiaticum negative-stranded RNA virus 1(FaNSRV1)	MZ969066
**92**	unassigned	-ssRNA	Fusarium poae negative-stranded virus 1	LC150618
**93**	unassigned	-ssRNA	Fusarium poae negative-stranded virus 2	LC150619

Supplementary Table S2. The family Hypoviridae includes eight genera and 55 species.

Genus	No.	Virus protein for alignment	NCIB Accession number	Year
Alphahypovirus	1	Fusarium sacchari hypovirus 1 isolate LZ1	this study	2024
		Fusarium sacchari hypovirus 1 isolate LZ8	this study	2024
		Fusarium sacchari hypovirus 1 isolate LZ14	this study	2024
		Fusarium sacchari hypovirus 1 isolate FS18	this study	2024
		Fusarium sacchari hypovirus 1 isolate LZ12	this study	2024
		Fusarium sacchari hypovirus 1 isolate FSA1	this study	2024
		Fusarium sacchari hypovirus 1 isolate FS3	this study	2024
		Fusarium sacchari hypovirus 1 isolate FS66	this study	2024
		Fusarium sacchari hypovirus 1 isolate FS65	this study	2024
		Fusarium sacchari hypovirus 1 strain FZ06	MN295969	2020
		Fusarium sacchari hypovirus 1 isolate FS1	this study	2024
		Fusarium sacchari hypovirus 1 isolate FS9	this study	2024
	2	Wuhan insect virus 14	KX883007	2017
	3	Trichoderma asperellum hypovirus 1	MK279475	2019
	4	Alternaria alternata hypovirus 1	MK189193	2019
	5	Fusarium graminearum hypovirus 1	MK279472	2019
	6	Fusarium sambucinum hypovirus 1	LC596823	2022
	7	Bipolaris oryzae hypovirus 1	MH316122	2019
	8	Cryphonectria hypovirus 1	M57938	1995
	9	Cryphonectria hypovirus 2	L29010	1994
	10	Botrytis cinerea hypovirus 2	MN617169	2021
	11	Macrophomina phaseolina hypovirus 1	KP900893	2016

	12	Macrophomina phaseolina hypovirus 2	MT062425	2020
	13	Pestalotiopsis fici hypovirus 1	OP441373-OP441375	2022
	14	Trichoderma harzianum hypovirus 2	MW863664	2021
	15	Cercospora beticola hypovirus 1	MZ546195	2022
**Zetahypovirus**	16	Sclerotinia sclerotiorum hypovirus 6	MH766501	2018
**Deltahypovirus**	17	Beihai_hypolike_virus_1	KX883006	2017
	18	Beihai_sipunculid_worm_virus_6	KX883005	2017
**Epsilonhypovirus**	19	Agaricus bisporus virus 2	KY357507	2017
	20	Entoleuca hypovirus 1	MF375885	2018
	21	Rosellinia necatrix hypovirus 2	LC333733	2018
	22	Sclerotinia homoeocarpa hypovirus 1	MK279473	2023
	23	Rosellinia necatrix hypovirus 1	LC318482	2018
	24	Fusarium graminearum hypovirus 2	KP208178	2015
	25	Fusarium langsethiae hypovirus 1	KY120321	2016
	26	Fusarium poae hypovirus 1	LC150612	2018
**Betahypovirus**	27	Trichoderma harzianum hypovirus 1	MN172262	2019
	28	Cryphonectria hypovirus 4	AY307099	2019
	29	Setosphaeria turcica hypovirus 1	MK279474	2019
	30	Fusarium oxysporum dianthi hypovirus 2	MN176979	2020
	31	Valsa ceratosperma hypovirus 1	AB690372	2012
	32	Phomopsis longicolla hypovirus	KF537784	2014
	33	Cryphonectria hypovirus 3	AF188515	2000
	34	Botrytis cinerea hypovirus 1	MG554632	2018
	35	Botrytis cinerea hypovirus 3	MN617170	2021

	36	Sclerotinia sclerotiorum hypovirus 1	JF781304	2011
	37	Sclerotinia sclerotiorum hypovirus 1-A	MF444220	2018
	38	Botryosphaeria dothidea hypovirus 1 (BdHV1/SXD111)	OR387868	2023
	39	Colletotrichum camelliae hypovirus 1	OR077707	2023
**Thetahypovirus**	40	Botrytis cinerea hypovirus 5	MT157414	2021
	41	Sclerotinia sclerotiorum hypovirus 2	MH347276	2019
	42	Sclerotium rolfsii hypovirus 1	MH037014	2018
	43	Botrytis cinerea hypovirus 4	MN617171	2022
	44	Rhizoctonia solani hypovirus 1	MK558259	2019
**Gammahypovirus**	45	Sclerotium rolfsii hypovirus 7	MH766502	2018
	46	Sclerotium rolfsii hypovirus 5	MH766500	2018
	47	Sclerotium rolfsii hypovirus 4	MH766499	2018
	48	Sclerotium rolfsii hypovirus 2	MH766497	2018
	49	Sclerotium rolfsii hypovirus 3	MH766498	2018
**Etahypovirus**	50	Sclerotium rolfsii hypovirus 8	MH766503	2018
**unassigned**	51	Rhizoctonia zeae hypovirus 1	OQ559666	2023
	52	Rhizoctonia zeae hypovirus 2	OQ559672	2023
	53	Alternaria dianthicola hypovirus 1	ON843755	2022
	54	Sclerotinia sclerotiorum hypovirus 9	MW454884	2021
	55	Erysiphe necator associated hypovirus 2	MN558694	2021

Supplementary Table S3. Primers used in this study.

**Primers for amplifying the full length of FsHV1**			
	Primer Name	Sequence (5’→ 3’)	Product size
**5'-RACE**	3'-GSP1	GGAGTCTTCCAATCAGAGCAG	250 bp
	3'-GSP2	GAACAAGATGCAAGCTCTAACTG	
**Fragment 1**	197-F	GGCACTTGTGCCGTTTACTC	1,530bp
	1627-R	CACCACCTGACAACACTTTGAG	
**Fragment 2**	1524-F	GCCACGAGGTATGTCAATCAC	1,554bp
	3078-R	CAACCACCAATCATCAGGGA	
**Fragment 3**	2991-F	CGGTGATGATTGGTTGCTTGA	494bp
	3485-R	GATAAGTAACCGCCACGAAGG	
**Fragment Defective**	FsHV1-DVF	GCACAATGGATGACACGAAGA	2,580bp
	FsHV1-DVR	GTAACCTGATTGAGACCTTCCG	
**Fragment 4**	5726-F	GTGAACCTAGTGCCAGTGCTTC	1,498bp
	7224-R	AGCACTACCACTCACCCAACC	
**Fragment 5**	7102-F	CATACTTGGAGGATTGGTGCA	1,611bp
	8713-R	CGGAGATATTCTTGAGCCATG	
**Fragment 6**	8564-F	CGAAAGACTCAATGATAACTGTGG	1,439bp
	10003-R	AGTCGTATTCTGGCTCGGTTA	
**Fragment 7**	9905-F	CAAGCGGTAATGGCAAGAA	1,569bp
	11474-R	CTTTCTTGAATAGCCTGGGACA	
**Fragment 8**	11370-F	CACTTACAACATGTGGACCGA	1,510bp
	12880-R	CGGCAGATTCTGGTTGATACAC	
**Fragment 9**	12486-F	TGCGTTCATGTTGGCTTGG	1,373bp
	13859-R	CATTCCTGGTGGCGTTGATAA	
**3'-RACE**	5'-GSP1	GCAGCCTAATAAGGTTCTCCGTGAATG	450bp
	5'-GSP2	GCAAAGCCCACGGGAGGAAATCCCAC	
**FsHV1 detection primer**			
**RdRp**	FsHV1-RdRpF1	GCTCAAGGTGCTGATGGTGTT	1,733bp
	FsHV1-RdRpR1	AGCGACATTGTAAGCGTGACC	
**Defective**	FsHV1-DefectF	GCACAATGGATGACACGAAGA	2,500bp or 800bp
	FsHV1-DefectR	GTAACCTGATTGAGACCTTCCG	
**Absolute quantitative primers**			
**FsHV1-Defective**	FsHV1-qDF	CGGTTCTGCTTACGCTTTTG	123bp
	FsHV1-qDR	AAGCAGGTTGATGCTCCAAT	
**FsHV1-RdRp**	FsHV1-RqF	CAGATGCAACGGCATTCGAC	102bp
	FsHV1-RqR	TGCCATTACCGCTTGGATGA	

**translation elongation factor 1-alpha (TEF-1α)**			
**EF-α**	EF1	ATGGGTAAGGARGACAAGAC	650 bp
	EF2	GGAAGTACCAGTSATCATGTT	
**DNA-directed RNA polymerase II largest subunit**			
**RPB1**	Fa	CAYAARGARTCYATGATGGGWC	1,600bp
	G2R	GTCATYTGDGTDGCDGGYTCDCC	
**DNA-directed RNA polymerase II second largest subunit**			
**RPB2**	5f2	GGGGWGAYCAGAAGAAGGC	1,800bp
	7cr	CCCATRGCTTGYTTRCCCAT	
	7cf	ATGGGYAARCAAGCYATGGG	
	11ar	GCRTGGATCTTRTCRTCSACC	

Supplementary Table S4. FsHV1-infected strains used in this study.

***Fusarium* species**	**Strain**	**Sample source**	**Location**	**Year of collection**
F. sacchari	FZ06	Sugarcane diseased leaves	Fuzhou, Fujian	2012
F. sacchari	FS65	Sugarcane diseased leaves	Fusui, Guangxi	2019
F. sacchari	FS66	Sugarcane diseased leaves	Fusui, Guangxi	2019
F. sacchari	FS9	Sugarcane diseased leaves	Fusui, Guangxi	2019
F. sacchari	FS18	Sugarcane diseased leaves	Fusui, Guangxi	2019
F. sacchari	FS1	Sugarcane diseased leaves	Fusui, Guangxi	2017
F. sacchari	FSA1	Sugarcane diseased leaves	Fusui, Guangxi	2019
F. sacchari	LZ14	Sugarcane diseased leaves	Longzhou, Guangxi	2020
F. sacchari	LZ8	Sugarcane diseased leaves	Longzhou, Guangxi	2020
F. andiyazi	FS3	Sugarcane diseased leaves	Fusui, Guangxi	2017
F. andiyazi	LZ12	Sugarcane diseased leaves	Longzhou, Guangxi	2020
F. solani	LZ1	Rhizosphere soil of sugarcane	Longzhou, Guangxi	2020

Supplementary Table S5. The efficiency of horizontal transmission of FsHV1.

VIrus	Species of the donor strain	Species of the recipient strains	Successful transmission/Total number
**FsHV1-FZ06**	*F. sacchari*	*F. sacchari*	18/30
**FsHV1-FS1**	*F. sacchari*	*F. sacchari*	9/30
**FsHV1-FS9**	*F. sacchari*	*F. sacchari*	10/30
**FsHV1-FS65**	*F. sacchari*	*F. sacchari*	8/30
**FsHV1-FS66**	*F. sacchari*	*F. sacchari*	4/30
**FsHV1-LZ8**	*F. sacchari*	*F. sacchari*	5/30
**FsHV1-FSA1**	*F. sacchari*	*F. sacchari*	8/30
**FsHV1-LZ14**	*F. sacchari*	*F. sacchari*	8/30
**FsHV1-FS18**	*F. sacchari*	*F. sacchari*	9/30
**FsHV1-FS3**	*F. andiyazi*	*F. sacchari*	11/30
**FsHV1-LZ12**	*F. andiyazi*	*F. sacchari*	4/30
**FsHV1-LZ1**	*F. solani*	*F. sacchari*	3/30

Supplementary Table S6. Amino acid variation between FsHV1-A1 and the other 11 FsHV1 isolates

Number	Position	Amino acids of FsHV1-A1	Amino acids of other 11 FsHV1 isolates
**1**	259	G	E
**2**	438	S	N
**3**	494	F	I
**4**	708	C	H or Y or R
**5**	740	G	E
**6**	1708	G	D
**7**	1874	L	P
**8**	1934	P	S
**9**	2093	I	V
**10**	2792	G	V
**11**	2815	F	I
**12**	2859	S	R
**13**	2890	T	N
**14**	3155	N	K
**15**	3453	R	Q
**16**	3193	G	D
**17**	4202	I	V
